# Genomic factors that shape craniofacial outcome and neural crest vulnerability in FASD

**DOI:** 10.3389/fgene.2014.00224

**Published:** 2014-08-07

**Authors:** Susan M. Smith, Ana Garic, Mark E. Berres, George R. Flentke

**Affiliations:** ^1^Department of Nutritional Sciences, University of Wisconsin–MadisonMadison, WI, USA; ^2^Department of Animal Sciences, University of Wisconsin–MadisonMadison, WI, USA

**Keywords:** fetal alcohol spectrum disorders, neural crest, sonic hedgehog, apoptosis, β-catenin, CaMKII, ribosome biogenesis

## Abstract

Prenatal alcohol exposure (PAE) causes distinctive facial characteristics in some pregnancies and not others; genetic factors may contribute to this differential vulnerability. Ethanol disrupts multiple events of neural crest development, including induction, survival, migration, and differentiation. Animal models and genomic approaches have substantially advanced our understanding of the mechanisms underlying these facial changes. PAE during gastrulation produces craniofacial changes corresponding with human fetal alcohol syndrome. These result because PAE reduces prechordal plate extension and suppresses sonic hedgehog, leading to holoprosencephaly and malpositioned facial primordia. Haploinsufficiency in sonic hedgehog signaling increases vulnerability to facial deficits and may influence some PAE pregnancies. In contrast, PAE during early neurogenesis produces facial hypoplasia, preceded by neural crest reductions due to significant apoptosis. Factors mediating this apoptosis include intracellular calcium mobilization, elevated reactive oxygen species, and loss of trophic support from β-catenin/calcium, sonic hedgehog, and mTOR signaling. Genome-wide SNP analysis links PDGFRA with facial outcomes in human PAE. Multiple genomic-level comparisons of ethanol-sensitive and – resistant early embryos, in both mouse and chick, independently identify common candidate genes that may potentially modify craniofacial vulnerability, including ribosomal proteins, proteosome, RNA splicing, and focal adhesion. In summary, research using animal models with genome-level differences in ethanol vulnerability, as well as targeted loss-and gain-of-function mutants, has clarified the mechanisms mediating craniofacial change in PAE. The findings additionally suggest that craniofacial deficits may represent a gene–ethanol interaction for some affected individuals. Genetic-level changes may prime individuals toward greater sensitivity or resistance to ethanol’s neurotoxicity.

## INTRODUCTION

It is indisputable that genetic factors modulate individual risk for alcoholism (see Edenberg and Foroud, 2013, for a recent review). For example, allelisms in alcohol and aldehyde dehydrogenases control blood alcohol levels and alcohol clearance rates. Allelisms in neurotransmitter receptors and their downstream effectors shape neuronal responses to alcohol reward, tolerance, and withdrawal. However, our understanding of how genetic factors might shape individual risk for fetal alcohol spectrum disorders (FASD) is less well defined. It is without question that prenatal alcohol exposure (PAE) is teratogenic; indeed, it is the most common teratogen exposure in western societies. However, there is appreciable variability in the severity of individual responses to PAE, even after controlling for the pattern, timing, and dose of alcohol intake. Some of this variance is due to environmental factors that include maternal nutrition and socioeconomic status. Evidence has been accumulating that genetic factors can also shape individual risk for FASD (Warren and Li, 2005). Concordance for FASD risk is greater in monozygotic than in dizygotic twins (Streissguth and Dehaene, 1993). The potential existence of genetic modifiers is strongly endorsed by animal models of FASD. Much of this animal work emphasizes morphological rather than behavioral outcomes. When ethanol exposure is held constant, genetic factors modulate the risk for cardiac, craniofacial, skeletal, and central nervous system defects in the developing offspring, and this is documented for both mammalian (primates, rats, mice) and non-mammalian (chick, zebrafish) developmental models (Goodlett et al., 1989; Gilliam and Kotch, 1996; Boehm et al., 1997; Chen et al., 2000; Debelak and Smith, 2000; Su et al., 2001; Loucks and Carvan, 2004; Downing et al., 2009, 2012a,b; Swartz et al., 2014). Because much of this work is summarized in other contributions to this volume, we will not further detail those findings here. Apart from allelisms that modulate alcohol clearance rates, the identities of genes that modify fetal vulnerability to FASD are not well characterized.

## ETHANOL INFLUENCES CRANIOFACIAL OUTCOMES IN FASD

Insights into how genetic factors may modify vulnerability to PAE are informed by an understanding of ethanol’s mechanisms of action. Unlike other teratogens, ethanol does not have a single receptor but instead affects cellular activity though structural interactions with diverse proteins that include ligand-gated ion channels, G-protein-coupled receptors, and intracellular signaling proteins (Howard et al., 2011). This diversity of targets enables ethanol to alter multiple signaling pathways and processes that are essential for normal development. Ethanol’s disruption of these pathways dysregulates cellular events that are central to morphogenesis, such as proliferative expansion, migration, differentiation, and survival. One well-characterized target of PAE is the developing face. PAE can cause facial characteristics that have diagnostic utility and these include a flattened midface, micrognathia, smooth philtrum, thin upper lip, and short palpebral fissures (Klingenberg et al., 2010). During development, the facial cartilage and bone are derived from the cranial neural crest, a stem cell population that originates at the neuroectoderm–ectoderm boundary during the process of neurulation (Sauka-Spengler and Bronner-Fraser, 2008). Shortly thereafter, cranial neural crest progenitors transform from an epithelial to mesenchymal phenotype, delaminate, and migrate laterally and ventrally to form the facial primordia (frontonasal, maxilla, and mandible), branchial arches (connective tissue of the thymus, thyroid, and cardiac outflow tract), cranial nerve elements, and melanocytes. At the same time, medial–lateral expansion of the underlying forebrain, or prosencephalon, further defines the size and relative positioning of the facial elements (frontonasal, maxilla, mandible, and hyoid). In humans, these events begin at day 17–18 post-fertilization, before the pregnancy is typically recognized. Animal models reveal that ethanol adversely affects many events of craniofacial development including neural crest induction, survival, migration, and expansion, as well as cranial midline development (Hassler and Moran, 1986; Cartwright et al., 1998; Dunty et al., 2001; Ahlgren et al., 2002; Rovasio and Battiato, 2002; Yan et al., 2010; Oyedele and Kramer, 2013). The mechanisms by which ethanol alters these events are the focus of a recent review (Smith et al., 2014), and the reader is referred to that article for a detailed discussion of those findings. This review instead emphasizes current knowledge of genetic influences upon craniofacial outcomes in PAE. It should be noted that women who abuse alcohol typically do so throughout pregnancy. Thus, it is likely that multiple mechanisms contribute to the craniofacial dysmorphology that partly typifies FASD.

## GENETIC BACKGROUND INFLUENCES CRANIOFACIAL OUTCOME IN FASD

Evidence that genome-level differences can influence craniofacial outcomes in PAE emerges largely from animal models. Mouse strains are especially powerful tools for this work, and numerous ethanol-sensitive and relatively resistant strains have been characterized. With respect to cranial development, the inbred strain C57BL/6J is considered ethanol sensitive, whereas DBA/J is considered ethanol resistant. Quantitative trait locus (QTL) mapping of the BxD recombinant lines derived from these strains established that vulnerability to ethanol’s teratogenicity has strong heritability and identified several large QTLs that may be contributory (Downing et al., 2012). The related inbred strains C57BL/6J and C57BL/6N also generate different facial dysmorphologies in response to PAE, although some of this may be due to differences in alcohol consumption (Anthony et al., 2010), as also found for the “U” and “N” rat strains (Wentzel and Eriksson, 2008). Non-mammalian embryos complement this rodent work because maternal influences are removed and direct embryonic effects can be investigated. The zebrafish strains AB, Ekkwill, and Tuebingen differ in their levels of cranial cell death, craniofacial dysmorphology, and overall survival in response to equivalent ethanol challenge (Loucks and Carvan, 2004). Commercial chick strains show similar variability, wherein broiler strains had greater cranial reductions compared with layer strains (Bupp Becker and Shibley, 1998), and layer strains themselves have differing facial apoptosis patterns and dysmorphologies in response to equivalent ethanol exposure (Debelak and Smith, 2000; Su et al., 2001). As detailed below, these genetic models have informed the mechanisms by which PAE disrupts craniofacial development.

## GENETIC INFLUENCES UPON HOLOPROSENCEPHALY IN FASD

The physical size and relationship of craniofacial structures are influenced by the underlying prosencephalon or forebrain. During gastrulation, anterior extension of the prechordal mesendoderm induces the overlying ectoderm to form neuroepithelium (Sauka-Spengler and Bronner-Fraser, 2008). Neural crest is specified at this ectoderm/neuroectoderm boundary. Simultaneous with this, the prechordal mesoderm induces sonic hedgehog (*shh*) within the neuroepithelial midline. Subsequently, *shh* activity at the prosencephalon midline drives expansion not only of the forebrain but also the overlying facial primordial. Thus, craniofacial development is intimately linked with brain induction and expansion.

Ethanol exposure at gastrulation disrupts midline formation and thereby craniofacial development. Ethanol exposure at gastrulation activates the *shh* suppressor protein kinase A and the increased protein kinase A activity downregulates *shh* at the embryo’s midline (Aoto et al., 2008). Ethanol-induced apoptosis within the anterior prechordal plate, as well as its reduced expansion, further limits neuroepithelial size and the neural crest induction field (Blader and Strahle, 1998; Aoto et al., 2008). Consequently, the prosencephalon expansion is reduced and the overlying facial primordia are malpositioned. Additionally, as shown in zebrafish, PAE also reduces cholesterol ester pools and thereby limits substrate availability for the covalent modification of the nascent N-terminal shh protein, which is necessary for the protein’s membrane association and *shh* signaling (Li et al., 2007). The reduced *shh* expression along the prosencephalon midline persists developmentally, as do reductions in additional inductive signals including *goosecoid*, *Foxa2*, and *Fgf8* (Li et al., 2007; Aoto et al., 2008; Hong and Krauss, 2012). Work in mice reveals that targeted ethanol exposure during these gastrulation-stage events generates the “classic” FAS face, including elongated upper lip, flattened philtrum, and reduced midface. These changes represent holoprosencephaly (Sulik, 1984; Lipinski et al., 2012) and are recreated in both mammalian and non-mammalian models of FASD (Sulik, 1984; Su et al., 2001; Carvan et al., 2004; Li et al., 2007; Aoto et al., 2008; Hong and Krauss, 2012; Lipinski et al., 2012). PAE at mouse e8.5 instead produces a distinct facial outcome that lacks these holoprosencephalic features, suggesting that criteria for recognizing facial dysmorphology in FASD may need expansion.

Genetic-level alterations within the *shh* signaling pathway increase vulnerability to facial dysmorphology in PAE. Mice that are haploinsufficient in *Shh*, *Gli2*, or *Cdon* generally have normal crania due to compensation from the remaining allele. However, ethanol exposure of these same heterozygotes at gestational day 7.0 (e7) causes holoprosencephaly, demonstrating this pathway’s mechanistic role in producing FASD facial changes (Hong and Krauss, 2012; Kietzman et al., 2014). It also suggests that haploinsufficiency in this pathway increases risk for ethanol-induced holoprosencephaly. In humans, holoprosencephaly is estimated to affect 1/16,000 live births and 1/250 conceptuses (Dubourg et al., 2007); thus, heterozygous carriers at risk for ethanol-induced damage may be more common in the general population than appreciated. It is possible that even mild ethanol exposure during this critical period increases the frequency and severity of human holoprosencephalic disorders.

## GENETIC INFLUENCES UPON NEURAL CREST APOPTOSIS IN FASD

Ethanol also alters craniofacial development through its induction of significant cell death within neural crest populations. This occurs at clinically relevant ethanol exposures from 20 to 100 mM (∼0.1 to ∼0.4 mg%). It has been documented for mouse, chick, and zebrafish models of PAE (Sulik et al., 1981; Cartwright et al., 1998; Carvan et al., 2004), suggesting that neural crest sensitivity is conserved across vertebrates and most likely also occurs in human exposure. This cell death is apoptotic, as the cells are positive for extracellular Annexin-V or terminal deoxynucleotidyl transferase (TUNEL), and their death is prevented by pretreating the cells with caspase inhibitors (Cartwright et al., 1998; Dunty et al., 2001; Carvan et al., 2004; Reimers et al., 2006). The apoptosis significantly reduces cranial neural crest numbers and contributes to craniofacial deficits (Sulik et al., 1981; Cartwright and Smith, 1995; Carvan et al., 2004; Garic et al., 2011; Flentke et al., 2014b). Multiple mechanisms contribute to this apoptosis, including the production of reactive oxygen species (Chen et al., 2013), generation of intracellular calcium transients (Debelak-Kragtorp et al., 2003), and the loss of tropic support from β-catenin (Flentke et al., 2011) and/or from sonic hedgehog (Ahlgren et al., 2002). The details of these mechanisms have been recently reviewed (Smith et al., 2014).

Genetic models offer insights into the mechanisms mediating this apoptosis. For example, neural crest populations from the mouse strain C56BL/6J have much greater apoptosis than do ICR neural crest at equivalent ethanol exposures (50–200 mM; Chen et al., 2000). The membrane content of GM1 ganglioside is enriched in ICR cells compared with those from C57BL/6J, and addition of GM1 ganglioside attenuates ethanol’s damage, suggesting that differences in GM1 content may affect ethanol vulnerability. Screens of zebrafish mutants have identified multiple genes that influence craniofacial outcomes in ethanol exposure including *pdgfra*, *plk1*, *hinfp*, *mars*, *vangl2*, and *foxi1* (McCarthy et al., 2013; Swartz et al., 2014). These are all thought to be loss-of-function mutations and all worsened the craniofacial dysmorphology in response to ethanol. At least two of these, *pdgfra* and *plk1* loss-of-function, also enhance apoptosis within ethanol-treated cranial regions. Importantly, PDGFRA also could be linked to craniofacial defects in individuals with FASD (McCarthy et al., 2013). It should be noted that loss-of-function of additional craniofacial genes did not modify the risk for ethanol-induced facial defects in this model, including *cyp26b1, gata3, smad5, smoothened, mitfa,* and *neurog1*, among others. This endorses that ethanol’s mechanism is specific rather than generalized.

## NEURAL CREST APOPTOSIS – INSIGHTS FROM THE CHICK MODEL

**FIGURE 1:**
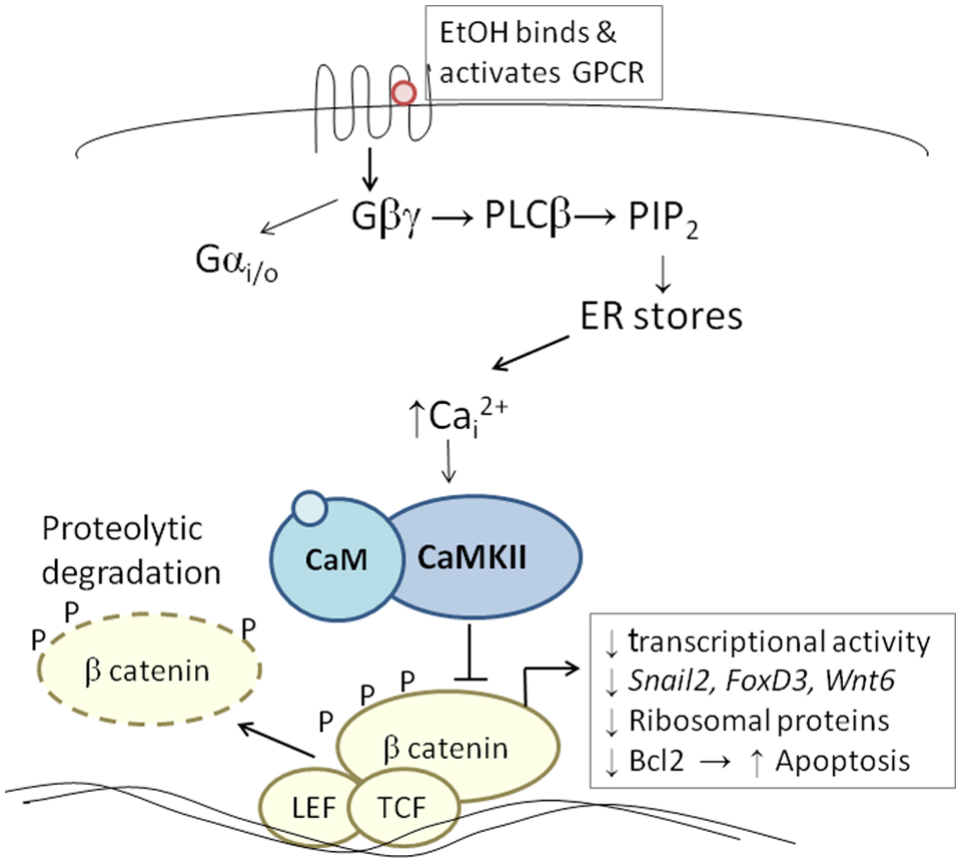
**Diagram of the calcium/β-catenin pathway by which ethanol causes the apoptosis of cranial neural crest populations, as deciphered for chick embryos having 1–10 somites; additional details in text.** Ethanol’s interaction with a G-protein-coupled receptor of unknown identity activates a calcium transient that originates from Gβγ, PLCβ, and phosphoinosityl phosphate release. The calcium transient stimulates calmodulin and CaMKII. CaMKII phosphorylates and thereby destabilizes β-catenin, abrogating the latter’s transcriptional activity. This removes trophic support from neural crest including genes critical for neural crest development (*snai2*, *FoxD3*, *Wnt6*). Accompanying reductions in bcl2 and ribosomal proteins may also be contributory.

**FIGURE 2:**
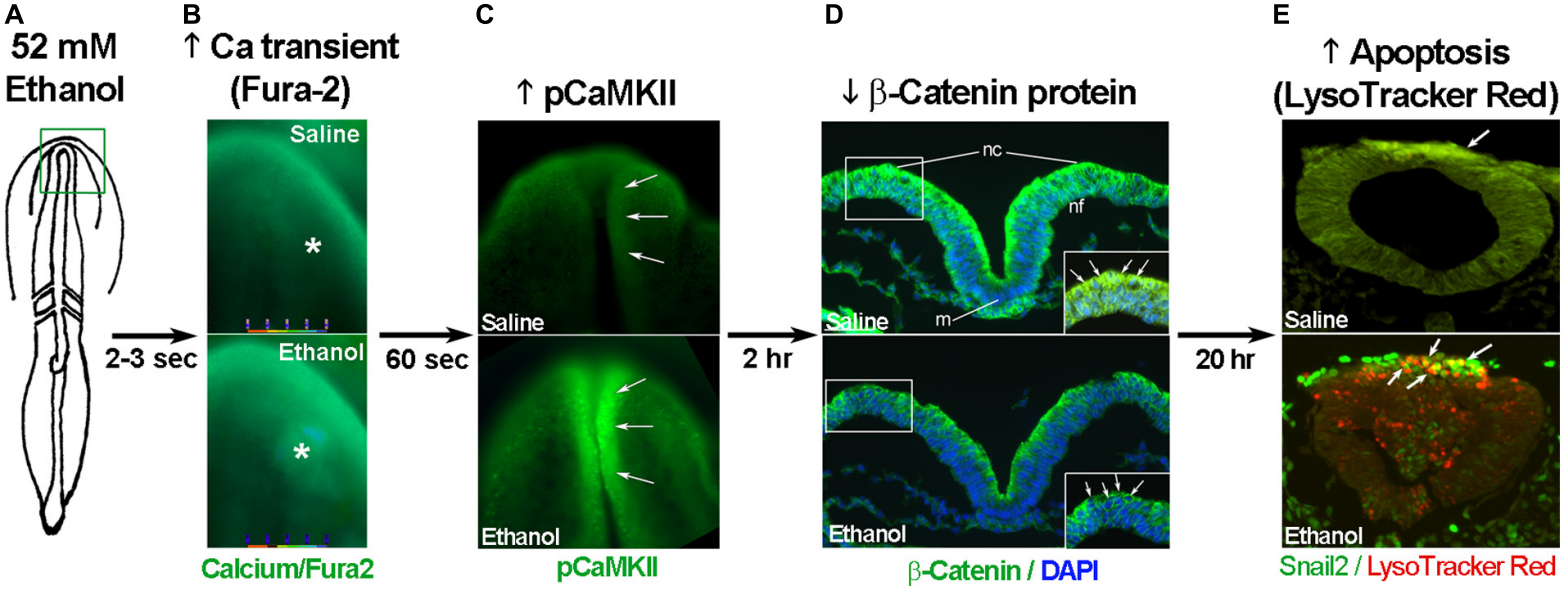
**Key events in ethanol-induced apoptosis of chick cranial neural crest. (A)** Diagram of chick embryo having 3 somites (stage 8-). **(B)** Exposure to 52 mM ethanol instigates the mobilization of intracellular calcium stores (*) within the early headfold (boxed region) as quantified using Fura2. Shown is dorsal view with anterior to the top. **(C)** The calcium transient selectively activates CaMKII within the anterior neural folds including neural crest (arrows) as detected using antibody directed against phospho-CaMKII (green signal, arrows). A dorsal view of the headfolds is depicted, anterior to the top. **(D)** Among other targets, CaMKII phosphorylates and destabilizes β-catenin protein (green signal at green arrows in boxed region) within the dorsal neural folds enriched in neural crest. Shown is a transverse section, dorsal at top, through the headfold of embryos having three somites; blue indicates DAPI-stained nuclei. **(E)** Subsequently, there is significant apoptosis (red signal) within ethanol-exposed dorsal neuroprogenitors of the hindbrain including neural crest (strong green signal), detected using antibody against the neural crest marker *snail2*. The saline-treated control hindbrain displays little cell death. Shown is a transverse section through rhombomere 4, which normally lacks appreciable cell death, of embryos having 16–18 somites; dorsal roof is at the top. Chick embryos normally have a low-level green autofluorescent background.

The developing chick embryo is advantageous for neural crest investigations due to its ready accessibility and ease of experimental manipulation. Much of the mechanism that governs neural crest apoptosis within this ethanol exposure model is now understood and this pathway is shown in **Figure 1**. In these cells, ethanol interacts with a G-protein-coupled receptor of yet-unknown identity and having a binding pocket that accommodates aliphatic alcohols from C1 through C5 (Garic-Stankovic et al., 2005, 2006). Alcohol binding stimulates G-protein signaling in which a pertussis toxin-sensitive Gβγ dimer activates phospholipase Cβ, likely the β4 isozyme that is expressed in neural crest (Garic-Stankovic et al., 2005). Within seconds of ethanol exposure, the resulting production of phosphoinositides initiates calcium mobilization from intracellular stores and the capacitative entry of extracellular calcium (**Figures 2A,B**; Debelak-Kragtorp et al., 2003; Garic-Stankovic et al., 2005). This calcium transient stimulates calmodulin and, within 1 min of ethanol exposure, the calmodulin-dependent kinase CaMKII is activated within neural crest and neuroprogenitors within the dorsal headfolds (**Figure 2C**, Garic et al., 2011). This action of ethanol is specific to these cells and CaMKII is not activated in ventral neural populations or in the presomitic mesoderm. CaMKII activation converts the short-lived calcium transient into a longer lived effector of neuroprogenitor fate, as CaMKII phosphorylates a number of downstream proteins within the cell. The induction of an intracellular calcium transient and CaMKII activity is essential and sufficient to produce neural crest apoptosis (**Figure 2E**).

Trophic support for early neural crest progenitors is provided by canonical Wnt signaling and its transcriptional effector β-catenin, which interacts with TCF/LEF proteins to induce gene expression (Kohn and Moon, 2005; MacDonald et al., 2009). Transcriptionally active β-catenin is indispensible for neural crest survival (Brault et al., 2001). Its transcriptional activity is negatively regulated through phosphorylation, which targets β-catenin for ubiquitination and proteolytic degradation. Ethanol’s calcium transient destabilizes nuclear β-catenin within 2 h of ethanol addition (**Figure 2D**) and significantly reduces its transcriptional activity, as measured using TopFlash reporter constructs and quantitation of known Wnt target genes (Flentke et al., 2011). β-Catenin over-expression in ethanol-treated neural crest is sufficient to rescue their survival and prevent their apoptosis (Flentke et al., 2011), and agents that sequester calcium similarly stabilize β-catenin in ethanol’s presence. Calcium is known to destabilize transcriptional β-catenin through multiple mechanisms including direct phosphorylation by protein kinase C, cleavage by calpain proteases, and indirectly through CaMKII phosphorylation of TCF/LEF. The GSK3β and JNK kinases can also directly phosphorylate and destabilize β-catenin (Kohn and Moon, 2005). Of those known effectors, only CaMKII inhibition stabilizes β-catenin and its transcriptional activity in ethanol-treated neural crest (Flentke et al., 2014a). Inhibiting other β-catenin effectors does not affect the protein’s stability or cell survival in ethanol-treated cells. Moreover, CaMKII directly phosphorylates β-catenin at three evolutionarily conserved and previously uncharacterized sites at T332, T472, and S552. Thus, β-catenin is a novel target for CaMKII’s kinase activity. Blocking any of the above steps within ethanol-exposed cells, using small molecules or targeted misexpression of loss/gain-of-function mutants within this pathway, fully prevents the apoptosis triggered by ethanol exposure (Debelak-Kragtorp et al., 2003; Garic-Stankovic et al., 2005; Flentke et al., 2011, 2014a; Garic et al., 2011).

Chick neural crest is not the only embryonic cell population in which an ethanol-induced calcium transient initiates apoptosis. Key elements of this pathway (calcium release, CaMKII activation **Figure 2E**) also mediate ethanol-induced apoptosis within zebrafish neural crest progenitors from equivalent developmental stages (Flentke et al., 2014b). Ethanol similarly invokes a pro-apoptotic, intracellular calcium transient within the gastrulating mouse, as well as in human cytotrophoblast cells and in mouse cerebellar neurons (Kilburn et al., 2006; Kouzoukas et al., 2013; Bolnick et al., 2014). Thus, this pro-apoptotic mechanism of ethanol’s action appears to be evolutionarily conserved and occurs in diverse cell lineages, at least within the embryo and fetus. Ethanol also invokes this same phosphoinositide-stimulated calcium transient in the mouse morula/blastocyst; however, it does not cause apoptosis and instead stimulates pathways that govern implantation and proliferative expansion (Winston and Maro, 1995). This suggests that how cells interpret ethanol’s calcium transient is lineage dependent.

## GENOMIC FACTORS MODIFY CALCIUM-MEDIATED NEURAL CREST APOPTOSIS

The serendipitous discovery of ethanol-sensitive and -resistant chicken strains provided novel insights into the mechanisms of this ethanol-mediated apoptosis. Layer flocks in the authors’ poultry facility are replaced annually and one such exchange revealed that the birds’ genetic background affects neural crest vulnerability to ethanol-induced apoptosis. As with inbred mice, commercial layer strains reproducibly fall along a continuum of ethanol responses. Some strains, such as Hy-Line W98 and W36, display high levels of neural crest apoptosis and a pronounced craniofacial dysmorphology, whereas other strains have little cell death and a relatively normal face (Debelak and Smith, 2000; Su et al., 2001). Vulnerability to ethanol-induced cardiac defects is similarly shaped by genetics (Cavieres and Smith, 2000). The ethanol content of eggs and embryos is equivalent and cannot account for this differential vulnerability. Because commercial chick strains are derived from hybrid crosses of sib-grandparent stocks, it is impractical to identify potential loci using traditional genetic breeding approaches. We therefore turned to deep DNA/RNA sequencing to characterize the transcriptomes of ethanol-sensitive and -resistant strains. The *Gallus gallus* genome was among the first to be sequenced and it is sufficiently annotated for detailed genetic analysis.

**FIGURE 3:**
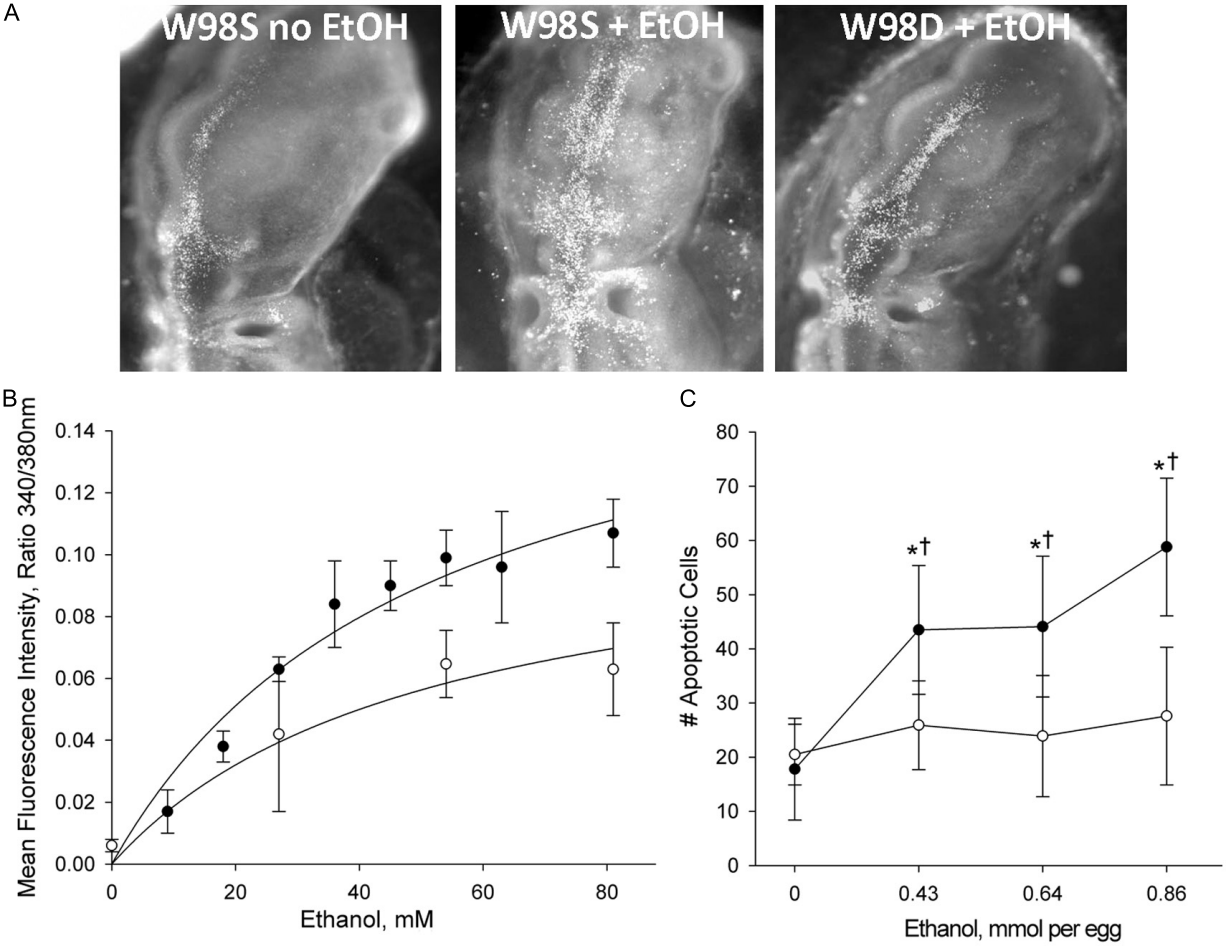
**Chick lines of the W98 background differ in their sensitivity to ethanol-induced apoptosis. (A)** There is appreciably more ethanol-induced cell death (white dots) in neural crest and neuroprogenitors in line W98S as compared with ethanol-treated W98D. Cell death levels in the latter do not differ from that in saline-treated controls. Here, cell death is visualized using acridine orange, which detects apoptosis in this model; dorsal view is shown. **(B)** W98S embryos (•) mobilize greater intracellular calcium concentrations (quantified using Fura2) in response to ethanol challenge than do W98D embryos (◦) at equivalent ethanol exposures. Their calcium release is dose-dependent. **(C)** W98S (•) has significantly more apoptotic cells than does W98D (◦) in response to equivalent ethanol exposures. Values are mean ± SD for 3–8 embryos/treatment; *p < 0.001 vs. 0 mM ethanol for within-strain comparison; ^†^*p* < 0.001 between W98S/W98D at equivalent ethanol dose.

This comparison was accomplished using a unique genetic resource, two related chicken lines of the Hy-Line W98 strain that were selected for multiple traits affecting egg production. These lines were maintained as distinct closed flocks for perhaps as many as 40 generations. Line W98S (so designated because it originated from Hy-Line’s Spencer, IA, facility) displays a robust calcium transient and high apoptosis in response to ethanol challenge (**Figure 3**; Garic et al., 2014), and it was utilized in many of the authors’ ethanol publications in the decade following 2000. In contrast, the related W98 line W98D (originating from Hy-Line’s Dallas Center, IA, facility) has ethanol-invoked calcium transients that are 30–40% lower than those of W98S at exposure to equivalent ethanol concentrations. Both strains achieve different plateau values, indicating that their differential response is not due to a shifted dose–response curve. Non-linear regression of these dose-response curves finds that their ethanol-induced calcium responses share similar Kds (51 mM vs. 55 mM) and different maxima (**Figure 3B**; Garic et al., 2014). This suggests that their neural crest progenitors possess ethanol-binding sites with similar affinities and that they differ in transduction of the ethanol signal.

**FIGURE 4:**
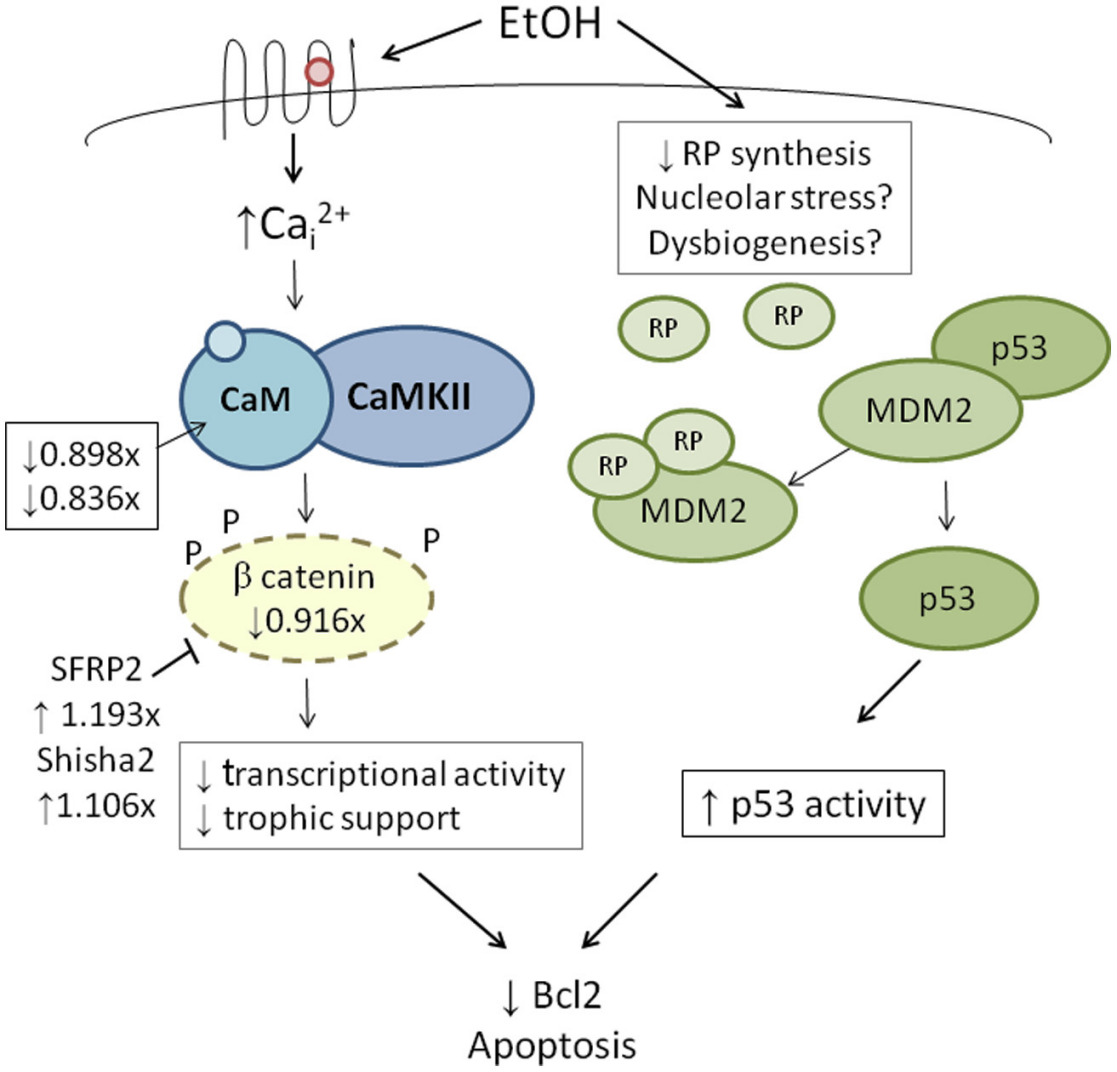
**Integration of genetic findings with known ethanol-induced signals in neural crest.** The left panel shows how the expressions of genes that participate in ethanol’s apoptosis are altered in within ethanol-sensitive W98S as compared against ethanol-resistant W98D. Fold-changes in expression are presented. The right panel proposes a mechanism by which ribosome dysgenesis could interact with MDM2 to stimulate p53-mediated apoptosis. In response to cellular stress such as ethanol, a decrease in ribosome synthesis leads to ribosome proteins interacting with MDM2. MDM2 no longer silences p53 and permits p53-mediated activities, including apoptosis, to increase within cells.

High-throughput transcriptome sequencing (RNA-Seq) of neural crest-enriched headfolds from W98S and W98D identified genomic difference that might influence ethanol sensitivity. Cells were not exposed to ethanol, so as to identify baseline differences that might potentially shape calcium and/or β-catenin signaling. The analysis identified 363 genes that are differentially expressed between W98S and W98D (Garic et al., 2014). Of these, 171 genes (47.1%) are increased in W98S and 192 genes (52.9%) are significantly decreased. Additionally, 18 genes within the Wnt/β-catenin signaling pathway have significantly differential expression. Importantly, the ethanol-sensitive W98S cells have significantly reduced expression of β-catenin itself (0.916-fold vs. W98D, *p* = 0.00588), as well as the two calmodulin isoforms that detect the calcium transient in these cells (CALM, 0.898-fold, *p* = 0.0170; CALM2, 0.836-fold, *p* = 0.060). W98S also has increased expression of two distinct Wnt/β-catenin antagonists, SHISA2 (1.106-fold, *p* = 2.34 × 10^-7^) and the secreted frizzled receptor protein SFRP2 (1.193-fold, *p* = 7.08 × 10^-8^), which is normally enriched in hindbrain neural crest progenitors fated for apoptosis (Ellies et al., 2002). Although these different expression levels represent transcripts and not protein, it suggests that cells derived from these two lines have foundational differences in how they perform Wnt/β-catenin-dependent signaling (**Figure 4**, left). In ethanol-sensitive W98S, the reduced β-catenin expression coupled with elevations in two canonical Wnt antagonists could dampen β-catenin’s transcriptional activity within its neural crest as compared with W98D. Similarly, their lower content of both calmodulin isoforms could alter their respective calcium signaling dynamics. Taken together, these changes could shift the ethanol dose–response curve such that W98D cells are buffered against β-catenin transcriptional losses, whereas W98S is more vulnerable to those losses. One caveat to this analysis is that these headfolds are composed of neural crest and neuroprogenitors and thus not all these genes are present in neural crest. However, Wnt signaling genes such as β-catenin and SFRP2 are restricted to neural crest at these stages and their identification in this model likely informs their cellular responses.

Additional analysis of this gene set using the Kyoto Encyclopedia of Genes and Genomes (KEGG) revealed 10 biologically characterized pathways with significantly differential representation between the two lines (**Table 1**; Garic et al., 2014). These pathways, in turn, largely contribute to two major cellular networks. In the first, four of these pathways mediate the flow of cellular information from nucleus to cytosol and included the spliceosome (*p* = 7.02 × 10^-8^), RNA transport (*p* = 0.00676), ribosome (*p* = 1.85 × 10^-47^), and protein processing in the endoplasmic reticulum (*p* = 0.00109) gene clusters. The second differentially represented network includes two pathways that govern energy generation and include metabolism (*p* = 0.00983) and oxidative phosphorylation (*p* = 1.10 × 10^-11^), and near significance for glycolysis/gluconeogenesis (*p* = 0.0530). Also differentially represented are KEGG pathways involving the cell cycle (*p* = 0.0140) and epithelial tight junctions (*p* = 0.00309).

**Table 1:** KEGG pathway enrichments in W98S vs. W98D.

KEGG	Identity	No. of genes	Significance
3010	Ribosomal	38	1.85 × 10^-47^
190	Oxidative phosphorylation	18	1.10 × 10^-11^
4260	Cardiac muscle	10	5.71 × 10^-9^
3040	Spliceosome	13	7.02 × 10^-8^
4141	Protein processing in ER	9	0.00109
4530	Epithelial tight junctions	6	0.00309
4114	Oocyte meiosis	6	0.00585
3013	RNA transport	7	0.00676
1100	Metabolism	25	0.00983
4110	Cell cycle	6	0.0140
10	Glycolysis/gluconeogenesis	18	0.0530
4145	Phagosome	5	0.0600

*Data from Garic et al. (2014).*

## NEURAL CREST AND RIBOSOME BIOGENESIS

**FIGURE 5:**
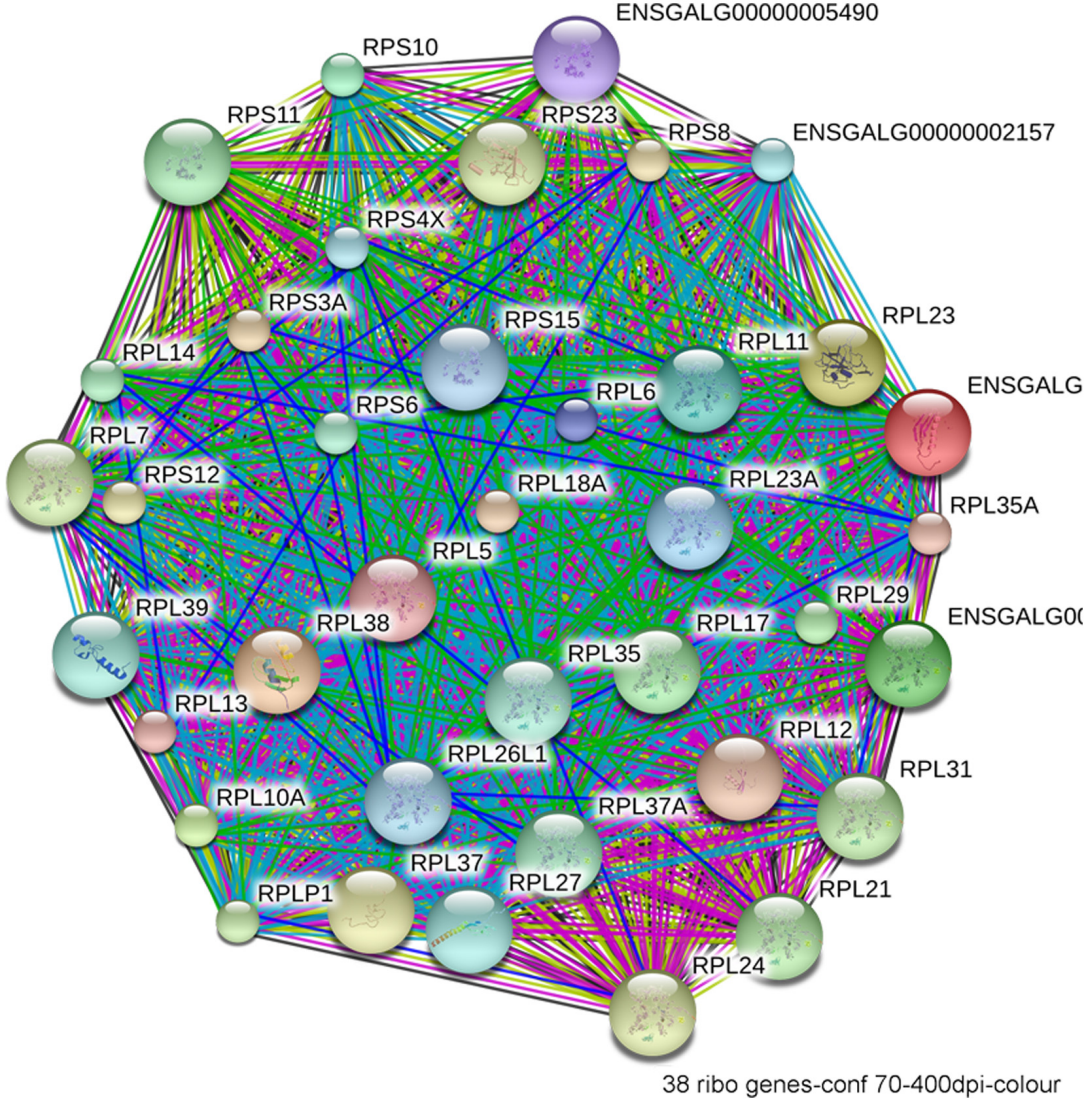
**Network interactions from the Database for Annotation, Visualization, and Integrated Discovery (DAVID) depict the relationships between the 38 ribosomal proteins that have significantly different expression between ethanol-sensitive W98S and ethanol-resistant W98D neural folds**.

The most compelling finding from the RNA-Seq comparison of ethanol-vulnerable/resistant neuroprogenitors is the significant differential enrichment of 38 genes that encode ribosome proteins. This includes 27 large and 11 small ribosomal subunit proteins (**Figure 5**), as well as two additional genes that participate in ribosome biogenesis, pescadillo (PES-1, 1.198-fold, *p* = 0.0145) and NSA2 (1.340-fold, *p* = 3.81 × 10^-6^). Of these ribosomal proteins, 25 were decreased and 13 increased in ethanol-sensitive W98S. Remarkably, ribosomal gene clusters were also significantly altered in two independent studies of ethanol exposure to mouse neural folds of comparable developmental stages to our chick embryos (Green et al., 2007; Downing et al., 2012b; see this volume). Ribosomal gene clusters additionally emerged from independent studies of ethanol-treated neuronal cultures (Rahman and Miles, 2001; Gutala et al., 2004). Perhaps, further supporting its importance, haploinsufficiency in the methionyl tRNA synthetase (*mars*), which donates the first amino acid to initiate protein translation, also heightens embryo sensitivity to ethanol-induced craniofacial deficits (Swartz et al., 2014). The repeated emergence of ribosomal protein clusters from multiple comparisons of ethanol-treated neuroprogenitors suggests that ribosomal activity may be an integral component of cellular ethanol responses.

Why are ribosomal proteins potentially relevant for neural crest apoptosis? Ribosome synthesis is complex (Granneman and Baserga, 2004). Ribosomes convert messenger RNA (mRNA) information into protein. Composed of four ribosomal RNAs (rRNA) and approximately 80 proteins, ribosomes bind mRNA, provide docking sites for codon recognition by amino acyl-transfer RNA, and catalyze the synthesis of nascent protein. The cell nucleolus denotes locations of polycistronic rRNA synthesis, processing, and ribosome assembly. The rRNA emerges as a single transcript that is then modified by small nucleolar RNAs (snoRNAs) and protein cofactors that mediate cleavage, methylation, and pseudouridylation of the rRNA. Ribosome biogenesis imposes a significant energy demand and, for rapidly proliferating cells, occupies as much as 80% of the energy budget because cells must disassemble the nucleolus and replenish their ribosomes with each mitotic division (Schmidt, 1999).

Given its high use of cellular resources, ribosome biogenesis has been recently recognized as an important sensor of cellular stress through its regulation of p53 activity (**Figure 4**, right; Kruse and Gu, 2009; Fumagalli and Thomas, 2011). The tumor suppressor protein p53 accumulates in response to stress to effect cell cycle arrest, apoptosis, DNA repair, and other processes. Interactions with the E3 ubiquitin ligase MDM2 silence p53 through MDM2-catalyzed ubiquitination and proteosomal destruction. When ribosome biogenesis is perturbed, as through reduced ATP availability or imbalanced ribosome protein content, ribosomal proteins such as RPL5, RPL11, RPL23, and RPS7 instead interact with MDM2 to suppress its ubiquitinase activity and thereby stabilize p53 and stimulate p53-mediated activities including cell cycle arrest and apoptosis.

Defects in ribosome biogenesis are clinically significant. Human ribosomopathies are not embryolethal and are associated with anemia, short stature, limb and heart defects, and, of relevance to this discussion, can feature significant craniofacial deficits including a flattened nasal bridge, micrognathia, epicanthal folds, cleft lip/palate, and altered palpebral fissues (Narla and Ebert, 2010; Fumagalli and Thomas, 2011). These facial changes echo those of FASD and suggest that cranial neural crest is especially sensitive to ribosome dysbiogenesis. Animal models confirm that loss-of-function mutations in ribosome proteins or effectors of ribosome biogenesis can cause cell cycle arrest and p53-mediated apoptosis within neural crest progenitors, the best described of these being Treacher-Collins syndrome (Trainor, 2010). Five of the differentially expressed ribosome proteins in W98S/D headfolds are known to regulate MDM2/p53 interactions (**Table 2**; RPL5, RPL11, RPL12, RPS15, RPL23). Additionally, nine of these ribosomal proteins are causative in the craniofacial ribosomopathy Diamond–Blackfan anemia (RPL5, RPL11, RPL26, RPL27, RPL35A, RPL36, RPS10, RPS15, RPS17). Additional attention to ribosome biogenesis arises from the analysis of these same cell populations 6 h following ethanol challenge, wherein the greatest gene cluster change again involves ribosome biogenesis (*p* = 2.2 × 10^-21^; Garic et al., 2014). Similar to the afore-mentioned reductions in *shh* signaling (Ahlgren et al., 2002) or in *Pdgfra* (McCarthy et al., 2013), genomic-level alterations in ribosomal proteins might disturb the balance of MDM2/p53 regulation that, in of itself, is insufficient to initiate apoptosis, but does so upon the additional stress of ethanol challenge (**Figure 4**). Indeed, interactions between mTOR and ribosome biogenesis control both cellular anabolism and decisions regarding p53 activity (reviewed in Shimobayashi and Hall, 2014), and it is tempting to speculate that these mTOR and ribosome/p53 findings have identified different aspects of the same mechanism. Given the established role for calcium/β-catenin signals in this model of ethanol-induced apoptosis, we additionally speculation that the combination of diminished β-catenin activity and ribosome dysbiogenesis may interact to stimulate these cells’ pro-apoptotic fate. Studies are underway to evaluate this hypothesis in detail.

**Table 2:** Differentially expressed RPs linked with human ribosomopathies.

Gene	Fold-change W98S/D	*p*-Value adjusted	MDM2/p53 effector	DBA-linked
RPL5	1.24	4.72 × 10^-19^	X	X
RPL11	0.88	0.00555	X	X
RPL12	0.75	4.63 × 10^-23^	X	–
RPL23	0.87	2.56 × 10^-6^	X	–
RPL26	0.89	0.00182	–	X
RPL27	0.83	1.27 × 10^-21^	–	X
RPL35A	0.85	5.01 × 10^-8^	–	X
RPL36	0.49	3.69 × 10^-34^	–	X
RPS10	0.93	0.0226	–	–
RPS15	1.17	1.67 × 10^-5^	X	X
RPS17	0.765	6.43 × 10^-13^	–	X

*Adapted from Narla and Ebert (2010), Fumagalli and Thomas (2011), and Garic et al. (2014).*

## ENERGY METABOLISM AND APOPTOSIS

**FIGURE 6:**
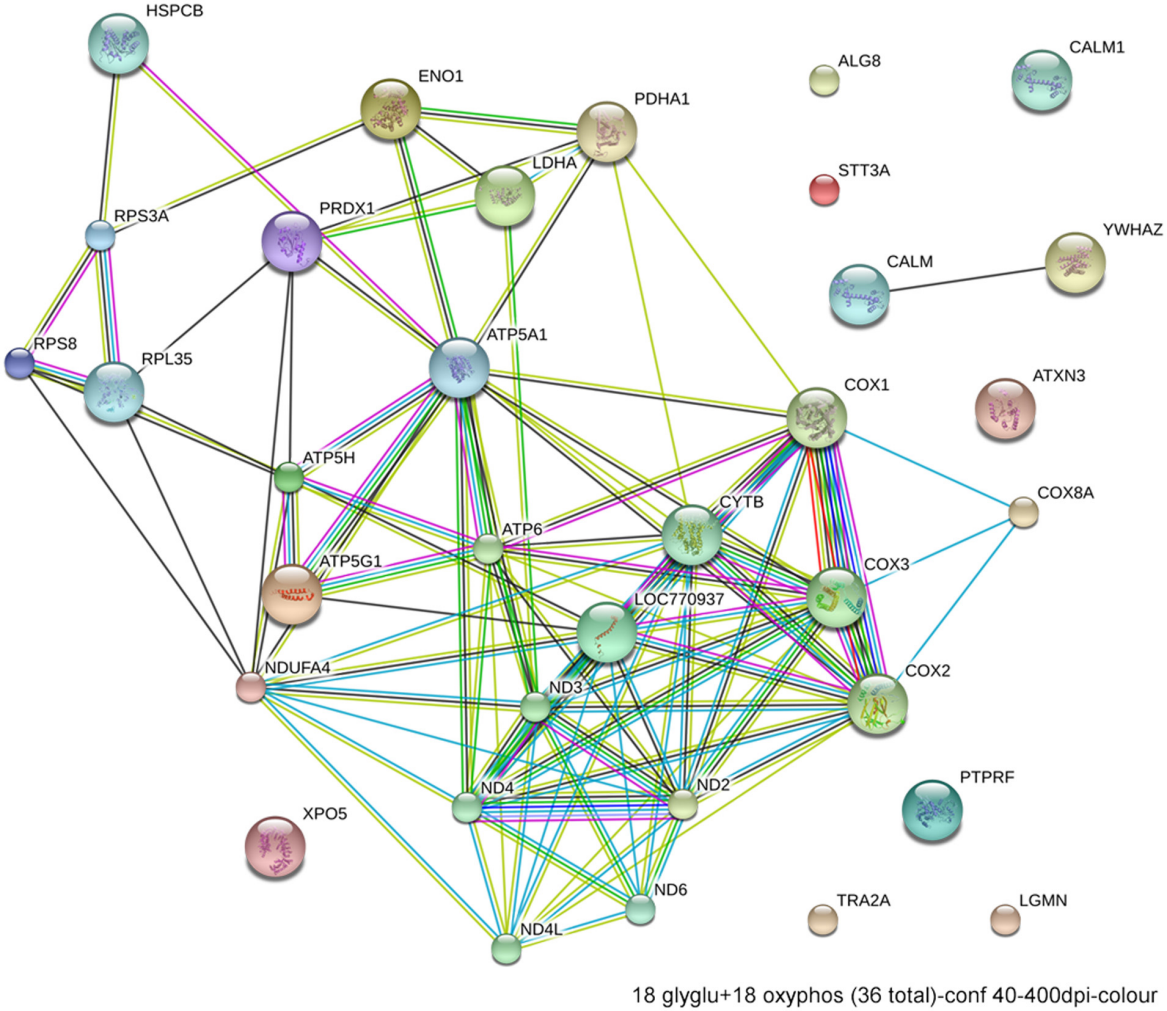
**Network interactions from DAVID depict the relationships between the 18 glycolytic and 18 oxidative phosphorylation genes that have significantly different expression between ethanol-sensitive W98S and ethanol-resistant W98D neural folds**.

Excessive ethanol suppresses energy metabolism through the competition for cellular reducing equivalents, through inhibition of lipolysis, and through direct effects upon mitochondrial activity (Bunout, 1999). Embryos have a high energy demand due to their obvious anabolic and pro-proliferative state. The avian embryo’s primary energy source is triglyceride β-oxidation and thus oxidative phosphorylation; unlike mammals, yolk-bearing embryos can convert acetyl-CoA subunits into glucose via gluconeogenesis. Ethanol-vulnerable cells from W98S had significant up-regulation of multiple components of oxidative phosphorylation including many proteins in complex I/NADH dehydrogenase, cytochrome c oxidase, and the ATP synthase (**Figure 6**). However, several components of the cytochrome c reductase and cytochrome c oxidase were significantly lower compared with W98D. It is possible that these changes altered the metabolic flux within W98S relative to W98D, but unfortunately those embryos are no longer available to test this hypothesis. These metabolic differences might also be linked to the ribosomal changes between these strains, given the high energy cost of ribosome biogenesis and its role as a cellular stress sensor. It is likely that these differences in energy metabolism resulted from commercial pressures during line selection, because efficiency in nutrient utilization and feed conversion are desirable agricultural traits to improve growth and production. Similarly, the emphasis on improved growth may have also created efficiencies in ribosome biogenesis. Given the importance of energy generation for the rapidly growing embryo, relative differences in energy metabolism may have influenced cellular vulnerability to ethanol-induced stress.

## SUMMARY

In summary, genetic approaches have informed the multiple mechanisms by which ethanol disrupts craniofacial morphogenesis. The use of haploinsufficient mice has demonstrated how PAE suppresses shh signaling and generates holoprosencephalic features when exposure occurs during gastrulation. Zebrafish mutants implicate additional mechanisms through effects upon mTOR signaling and cell cycle regulation. Transcriptome sequencing of both mouse and avian embryos with differing ethanol sensitivities has further identified novel potential mechanisms including energy metabolism and/or ribosome biogenesis; in turn, these could be related to each other as well to contributions from mTOR. That ribosomal dysregulation also emerged from multiple, independent analyses of ethanol responsive genes in mouse neural folds make ribosome biogenesis a strong candidate to modify and contribute to ethanol’s damage. Our findings suggest that genetic-level differences in neural crest vulnerability to PAE may partially explain why only a percentage of ethanol-exposed pregnancies exhibit a postnatal craniofacial dysmorphology. Next-generation sequencing offers a rapid and comparatively affordable approach to identify additional genomic candidates that modify craniofacial responses to ethanol. Together with advances in epigenetics, this research will greatly advance our understanding of how genes and environment interact to shape individual outcomes in FASD.

## Conflict of Interest Statement

The authors declare that the research was conducted in the absence of any commercial or financial relationships that could be construed as a potential conflict of interest.
